# Survival of people with cystic fibrosis in Australia

**DOI:** 10.1038/s41598-022-24374-4

**Published:** 2022-11-17

**Authors:** Rasa Ruseckaite, Farhad Salimi, Arul Earnest, Scott C. Bell, Tonia Douglas, Katherine Frayman, Lucy Keatley, Susannah King, Tom Kotsimbos, Peter G. Middleton, Sue Morey, Siobhain Mulrennan, Andre Schultz, Claire Wainwright, Nathan Ward, Peter Wark, Susannah Ahern

**Affiliations:** 1 Department of Epidemiology and Preventive Medicine, Monash University, Melbourne, VIC Australia; 2 Thoracic Medicine, Adult Cystic Fibrosis Centre, The Prince Charles Hospital, Brisbane, QLD Australia; 3 Children’s Health Research Centre, The University of Queensland, Brisbane, QLD Australia; 4 Translational Research Institute, Brisbane, QLD Australia; 5 Faculty of Medicine, Clinical Unit, University of Queensland, Brisbane, QLD Australia; 6 Department of Respiratory and Sleep Medicine, Royal Children’s Hospital, Melbourne, VIC Australia; 7 Respiratory Diseases Group, Murdoch Children’s Research Institute, Melbourne, VIC Australia; 8 Cystic Fibrosis Service, Department of Respiratory and Sleep Medicine, Westmead Hospital, Sydney, News South Wales Australia; 9 Nutrition Department, The Alfred, Melbourne, VIC Australia; 10 Discipline of Food, Dietetics and Nutrition, LaTrobe University, Melbourne, VIC Australia; 11 Department of Respiratory Medicine, The Alfred, Melbourne, VIC Australia; 12 Department of Respiratory Medicine, Sir Charles Gairdner Hospital, Perth, WA Australia; 13 Institute of Respiratory Health, University of Western Australia, Perth, WA Australia; 14 Perth Children’s Hospital, Perth, WA Australia; 15 Wal-Yan Centre for Respiratory Health, Telethon Kids Institute, University of Western Australia, Perth, Australia; 16 Cystic Fibrosis Service, Department of Respiratory and Sleep Medicine, Queensland Children’s Hospital, Brisbane, QLD Australia; 17 Physiotherapy/Department of Thoracic Medicine, Royal Adelaide Hospital, Adelaide, South Australia Australia; 18 Immune Health Programme, Hunter Medical Research Institute, University of Newcastle, Newcastle, NSW Australia

**Keywords:** Public health, Cystic fibrosis, Epidemiology

## Abstract

Survival statistics, estimated using data from national cystic fibrosis (CF) registries, inform the CF community and monitor disease progression. This study aimed to estimate survival among people with CF in Australia and to identify factors associated with survival. This population-based cohort study used prospectively collected data from 23 Australian CF centres participating in the Australian CF Data Registry (ACFDR) from 2005–2020. Period survival analysis was used to calculate median age of survival estimates for each 5-year window from 2005–2009 until 2016–2020. The overall median survival was estimated using the Kaplan–Meier method. Between 2005–2020 the ACFDR followed 4,601 people with CF, noting 516 (11.2%) deaths including 195 following lung transplantation. Out of the total sample, more than half (52.5%) were male and 395 (8.6%) had undergone lung transplantation. Two thirds of people with CF (66.1%) were diagnosed before six weeks of age or by newborn/prenatal screening. The overall median age of survival was estimated as 54.0 years (95% CI: 51.0–57.04). Estimated median survival increased from 48.9 years (95% CI: 44.7–53.5) for people with CF born in 2005–2009, to 56.3 years (95% CI: 51.2–60.4) for those born in 2016–2020. Factors independently associated with reduced survival include receiving a lung transplant, having low FEV_1_pp and BMI. Median survival estimates are increasing in CF in Australia. This likely reflects multiple factors, including newborn screening, improvement in diagnosis, refinements in CF management and centre-based multidisciplinary care.

## Introduction

Cystic fibrosis (CF) is the most common autosomal recessive-inherited life-limiting condition, affecting approximately 90,000 individuals worldwide^1^. People with CF require support from healthcare services from diagnosis onwards; and respiratory failure is the commonest cause of premature death^2^. Although life expectancy for people with CF has increased substantially, the disease continues to result in reduced life expectancy, poorer quality of life, and a large burden of care for people with CF, their families and health care providers^3^. Prognosis continues to improve due to advancements in CF care^4^, such as the availability of new inhaled antibiotics, newborn screening (NBS), *P. aeruginosa* eradication therapy, mucolytic treatment, better growth and nutrition, lung transplantation and lifetime multidisciplinary care in specialised CF centres^5^.

Although CF survival estimates have greatly improved globally, survival continues to be influenced by various individual factors^6^. A recent study of UK registry data demonstrated that male sex was associated with better survival, as was later diagnosis in adulthood, but only in non-F508del homozygotes. Survival did not differ by genotype among individuals diagnosed in early infancy^2^. In the recent study of Durda-Masny et al^7^, the shortest life expectancy was observed in adult patients with a severe mutation on both alleles, Forced Expiratory Volume in one second percent predicted (FEV_1_pp) < 40%, patients infected with extensively drug-resistant *P. aeruginosa*, and body mass index (BMI) < 18.5 kg/m^2^. Most of the deaths in these people occurred between 30 and 40 years of age.

Providing up-to-date estimates of survival is helpful for counselling people with CF and their families on life expectancy, planning social and healthcare needs, optimising educational and occupational opportunities, guiding genetic counselling, the development of new therapies and evaluating the effectiveness of health interventions. Survival data is important in the development of evidence-based guidelines and standards of care for CF management and workforce planning, including screening, monitoring and management of age-related co-morbidities and complications. Further, comparisons of survival internationally promote equitable global health outcomes. Using a standardised approach to data processing and survival calculations will provide greater confidence in international comparisons and in the identification of factors that may contribute to the observed differences^2,8^.

The objectives of this study were to use a standardised approach for estimating survival among patients participating in the Australian CF registry. Specific aims of this study were to:1) estimate median survival for Australian people with CF, 2) identify factors associated with survival, and 3) estimate median age of survival in successive 5-year cohorts beginning with the period 2005–2009 up until 2016–2020.

## Material and methods

### Data source

This population-based cohort study used prospectively collected Australian Cystic Fibrosis Data Registry (ACFDR) data from 2005 to 2020 inclusive. The available data spanned 1998–2020, but analyses were limited to a cohort eligible from January 2005 through July 2021 to reflect completeness of dates of clinic visits used in the analysis.

The ACFDR contains detailed demographic and clinical information about people with a confirmed diagnosis of CF, receiving clinical care at 23 CF centres in Australia^9^. It captures > 90% of all Australians with CF via enrolment in the registry, and at the end of 2020 there were 3,538 Australians diagnosed with CF whose records were registered with the ACFDR^9^.

Data including age, sex, age of diagnosis, symptoms at presentation, diagnosis by NBS/prenatal screening, date of transplantation, date and cause of death, as well as anthropometric measurements, lung function, microbiology, CF-related complications, and pancreatic status were collected. Data were censored at the time of transplantation.

A detailed description of the registry is provided elsewhere^9–11^.

### Definitions of key variables

Diagnosis was categorized as: those detected with meconium ileus symptoms; diagnosed before 6 weeks of age or by NBS/prenatal screening; diagnosed 6 weeks– < 2 years of age; 2–17 years and ≥ 18 years of age. Genotype was classified as F508del homozygous or other. Lung function, specifically the forced expiratory volume in one second (FEV_1_), was recorded in litres and the percent predicted (FEV_1_pp) was calculated using Global Lung Function Initiative (GLI) reference equations^12^. BMI was calculated using weight/height^2^. Adult individuals (≥ 18 years) were classified into BMI categories based on World Health Organization guidelines as underweight (< 18.5 kg/m^2^), adequate weight status (18.5–24.9 kg/m^2^), or overweight (≥ 25.0 kg/m^2^)^13^. Children were classified into BMI percentile categories based on Australian New Zealand CF Nutrition guidelines as underweight (< 10th percentile), adequate weight (10–85th percentile), or overweight (> 85th percentile)^14^. Pancreatic insufficiency (PI) was determined by pancreatic enzyme use at the first data entry into the registry. Missing values were included as a separate category in the regression models.

### Statistical analyses

Descriptive statistics were used to describe the study population. Demographic and clinical variables were summarised with categorical variables expressed as frequency and proportion and continuous variables summarised as mean, standard deviation (SD) and range.

Overall survival probability was estimated using Kaplan–Meier survival curve^15^. Period survival analysis was used to calculate median age of survival estimates over time^16^. Median age of survival was calculated for each 5-year window beginning with the period 2005–2009 and ending with 2016–2020. Univariate and multivariable Cox proportional hazard models with age as the underlying time were used to assess the associations between personal and clinical characteristics and mortality^17^.

Data were left truncated at the age on the 1 January of the year individuals were enrolled in the ACFDR and right censored at the date on which they were last seen. Death was defined as the event. Date of diagnosis was set at 30 days post date of birth for people with missing date of diagnosis. Any post-lung transplantation FEV_1_ and BMI measurements were removed from the analyses. The following personal and clinical characteristics were included in the model: sex (male vs female, time-independent variable), diagnosis category ((i) with meconium ileus symptoms, (ii) under < 6 weeks or by NBS/prenatal screening, (iii) 6 weeks– < 2 years, (iv) 2–17 years and (v) ≥ 18 years groups, time-independent variable), genotype (*F508del homozygous* or not, time-independent variable), pancreatic status (sufficient vs insufficient, due to uncertain quality of the earlier data, this variable was considered as time-independent), BMI (underweight, adequate weight status or overweight, categorical time-dependent variable), lung transplantation (binary, time-dependent variable), and FEV_1_pp (≥ 70, 40–69, or < 40), categorical time-dependent variable). FEV_1_ data were not recorded in those < 6 years of age.

All the analyses were performed in R version 4.1.1 and survival library 3.2 (https://www.R-project.org).

### Ethics approval

The study had ethics approval from the Alfred Health Human Research Ethics Committees, Melbourne, Victoria, Australia (Project Number HREC/16/Alfred/187). Informed consent was obtained from individuals and parents/guardians of those < 18 years of age, and for sites where local ethics committee required this, and an opt-out model was applied to the other sites where patients had the opportunity to contact the registry and opt out from the registry. All methods were carried out in accordance with relevant guidelines and regulations.

## Results

There were 4,601 people with CF observed over the period of 2005–2020 in the ACFDR. Table 1 shows basic demographic and clinical characteristics of the study sample.

**Table 1:** Demographic and clinical variables of the study population, 2005–2020.

	Survived, N (%)	Died, N (%)	Total, N (%)	*p*
	4,085 (88.8%)	516 (11.2%)	4,601 (100%)	
**Sex**				0.840^1^
Female	1,943 (47.6%)	243 (47.1%)	2,186 (47.5%)	
Male	2,142 (52.4%)	273 (52.9%)	2,415 (52.5%)	
**Diagnosis category**				< 0.001^1^
Diagnosed with meconium ileus	480 (11.8%)	48 (9.3%)	528 (11.5%)	
Age < 6 weeks or through NBS/Prenatal screening	2,747 (67.2%)	296 (57.4%)	3,043 (66.1%)	
Age 6 weeks–< 2 years	376 (9.2%)	97 (18.8%)	473 (10.3%)	
Age 2 – 17 years	325 (8.0%)	46 (8.9%)	371 (8.1%)	
Age ≥ 18 years	157 (3.8%)	29 (5.6%)	186 (4.0%)	
**F508del homozygous**				0.864^1^
No	2,223 (54.4%)	275 (54.1%)	2,498 (54.4%)	
Yes	1860 (45.6%)	233 (45.9%)	2,093 (45.6%)	
**Lung transplant**				< 0.001^2^
No	3,885 (95.1%)	321 (62.2%)	4,206 (91.4%)	
Yes	200 (4.9%)	195 (37.8%)	395 (8.6%)	
**Pancreatic exocrine status**				< 0.436^1^
Insufficient	2,836 (69.4%)	368 (71.3%)	3,204 (69.6%)	
Sufficient	972 (23.8%)	110 (21.3%)	1,082 (23.5%)	
Missing	277 (6.8%)	38 (7.4%)	315 (6.8%)	
**Mean FEV1pp across the lifetime**				< 0.001^2^
Not assessed, age < 6 years^3^	516 (12.6%)	3 (0.6%)	519 (11.3%)	
Mean (SD)	83.1 (21.0)	55.7 (19.7)	79.7 (22.7)	
Range	17.0–130.0	10.0–111.6	10.0–130.0	
Missing	138 (3.9%)	27 (0.6%)	165 (3.6%)	
**Last recorded BMI**^**4**^				< 0.001^1^
Underweight	182 (4.5%)	134 (26.0%)	316 (6.9%)	
Adequate weight status	2,670 (65.4%)	312 (60.5%)	2,982 (64.8%)	
Overweight	1,076 (26.3%)	47 (9.1%)	1,123 (24.4%)	
Missing	157 (3.8%)	23 (4.5%)	180 (3.9%)	
**Mean last recorded BMI**				
Adults (N)	2,359	462	2,821	< 0.001^2^
BMI, Mean (SD)	23.5 (4.9)	20.6 (3.5)	23.0 (4.8)	
Range	13.9–167.5	12.5–35.8	12.5–157.5	
Missing	89	21	110	
Children (N)	1,726	54	1,780	< 0.001^2^
BMI percentile, Mean (SD)	0.6 (0.3)	0.4 (0.3)	0.6 (0.3)	
Range	0.0–1.0	0.0–1.0	0.0–1.0	
Missing	68	2	70	

*BMI* body mass index, *FEV*_*1*_*pp* percent predicted forced expiratory volume in one second.

1 Pearson’s Chi-squared test, 2 Linear Model ANOVA, 3 Lung function not captured for those < 6 years of age, 4 Adult individuals (≥ 18 years) were classified into BMI categories based on World Health Organization guidelines as underweight (< 18.5 kg/m^2^), adequate weight (18.5–24.9 kg/m^2^), or overweight (≥ 25.0 kg/m^2^). Children were classified as underweight (< 10th percentile), adequate weight (10–85th percentile), or overweight (> 85th percentile).

There were more males (52.5%). Nearly half (45.6%) of all individuals with CF were F508del homozygous. Two thirds of people with CF (66.1%) were diagnosed < 6 weeks of age or by NBS/prenatal screening. Less than a third (23.5%) of the study population were pancreatic sufficient. Participants who survived were more likely to be diagnosed before 6 weeks of age, via NBS/prenatal screening (67.2% vs. 57.4%) and to have not received a transplant (95.1% vs. 62.2%).

**Figure 1:**
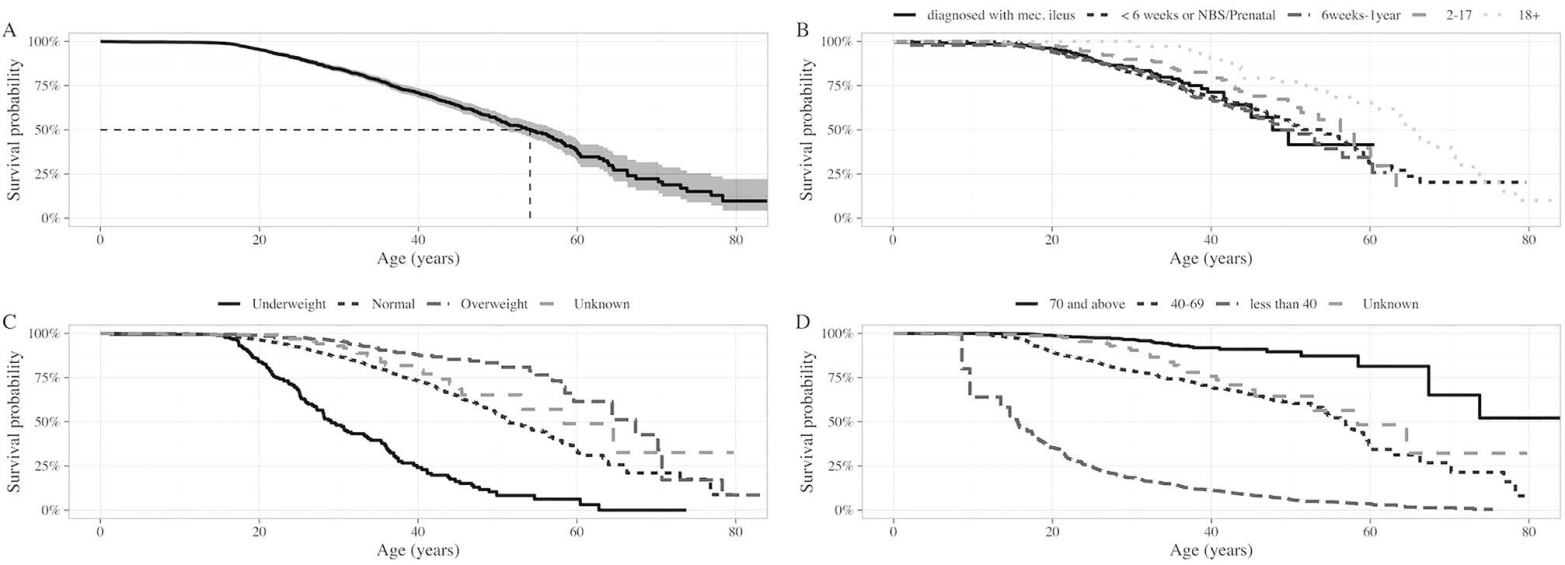
(**A**) Overall survival curve for people with cystic fibrosis, based on Kaplan–Meier estimates. (**B**) Probability functions depicting the age of people with cystic fibrosis by diagnosis category (with meconium ileus symptoms, under < 6 weeks or by NBS/prenatal screening, 6 weeks – < 2 years, 2–17 years and ≥ 18 years groups). (**C**) Probability functions depicting the age of people with CF by average lifetime lung function (FEV1 pp categories ≥ 70, 40–69, < 40). (**D**) Probability functions depicting the age of people with cystic fibrosis by BMI. Adult individuals (≥ 8 years) were classified into BMI categories based on World Health Organization guidelines as underweight (< 18.5 kg/m^2^), adequate weight (18.5–24.9 kg/m^2^), or overweight (≥ 25.0 kg/m^2^). Children were classified as underweight (< 10th percentile), adequate weight (10–85th percentile), or overweight (> 85th percentile).

Mean BMI for adults and BMI percentile for children were similar in survivors compared to non-survivors. Overall median survival age of individuals with CF in Australia was estimated as 54.0 years (95% CI: 51.0–57.4) (Fig. 1A). Survival analysis using the Kaplan–Meier method shows the survival of people with CF is almost 100% up to the age of 12 and then starts to gradually decline. Of those people followed to the end of 2020, 648 (14.1%) were aged 40 years or more or had reached > 40 years at age of death. At the time of the data analysis, 69 (1.5%) of those followed were aged > 60 years or had reached > 60 years at death. Of those aged > 60 years, 38 (55%) were diagnosed when they were adults.

The Kaplan–Meier curves varied significantly depending on individual characteristics such as age at diagnosis (*p* value of the log-rank score test = 0.001 (Fig. 1B), lung function (*p* value < 0.001) (Fig. 1C), and BMI (*p* value < 0.001) (Fig. 1D).

Table 2 displays the univariate and multivariable hazards ratios (HR) with 95% CIs, assessing the effects of the predictors of death.

**Table 2:** Univariate and multivariable Cox proportional hazard models for death adjusted for demographic and clinical characteristics (2005–2020).

	Univariate model		Multivariable model	
	Hazard ratio	95% CI	Hazard ratio	95% CI
**Sex**				
Female	1.00	–	1.00	–
Male	0.91	0.77 – 1.09	1.00	0.83 – 1.19
**Diagnosis category**				
Diagnosed with meconium ileus	1.00	–	1.00	–
Age < 6 weeks or through NBS/Prenatal screening	1.06	0.78 – 1.45	1.37	1.00 – 1.88*
Age 6 weeks– < 2 years	1.12	0.79 – 1.59	1.12	0.79 – 1.59
Age 2 – 17 years	0.74	0.49 – 1.12	0.85	0.56 – 1.29
Age ≥ 18 years	0.60	0.37 – 1.00*	0.88	0.52 – 1.50
**F508del homozygous**				
No	1.00	–	1.00	–
Yes	1.12	0.93 – 1.33	1.09	0.91 – 1.30
**Lung transplant**				
No	1.00	–	1.00	–
Yes	9.49	7.83 – 11.51**	3.56	2.84 – 4.47**
**Pancreatic exocrine status**				
Sufficient	1.00	–	1.00	–
Insufficient	1.33	1.07 – 1.64*	1.13	0.91 – 1.40
Missing	1.17	0.80 – 1.69	1.14	0.78 – 1.66
**FEV**_**1**_** pp**				
≥ 70	1.00	–	1.00	–
40–69	5.49	3.98 – 7.56**	4.60	3.33 – 6.36**
< 40	22.32	16.2 – 30.75**	9.77	6.84 – 13.96**
Missing	3.71	2.3 – 5.98**	4.95	1.76 – 13.88*
**BMI**^1^				
Adequate weight status	1.00	–	1.00	–
Underweight	4.42	3.6 – 5.42**	1.93	1.54– 2.41**
Overweight	0.41	0.3 – 0.56**	0.79	0.57 – 1.08
Missing	0.7	0.46 – 1.08	0.61	0.21 – 1.77

*BMI* body mass index, *FEV1pp* percent predicted forced expiratory volume in one second.

1 Adult individuals (≥ 18 years) were classified into BMI categories based on World Health Organization guidelines as underweight (< 18.5 kg/m^2^), adequate weight (18.5–24.9 kg/m^2^), or overweight (≥ 25.0 kg/m^2^). Children were classified as underweight (< 10th percentile), adequate weight (10-85th percentile), or overweight (> 85th percentile). * p < 0.05. **p < 0.001.

In the multivariable model which was adjusted for individual characteristics, no difference in survival between males and females was observed (HR 1.00, 95% CI: 0.83–1.19). Compared to those who were diagnosed with meconium ileus symptoms, the risk of death was 37% higher in those who were diagnosed aged < 6 weeks or by NBS/prenatal screening (HR 1.37, 95% CI: 1.00–1.88). This effect was observed in the multivariable model only. The risk of death was highest in those who underwent lung transplant (HR 3.56, 95% CI: 2.84–4.47). In the model adjusted for both demographic characteristics and clinical factors, the risk of death remained significantly higher in those with lifetime average FEV_1_pp of 40–69% (HR 4.60 95% CI 3.33–6.36) and with lifetime average FEV_1_pp of < 40% (HR 9.77, 95% CI 6.84–13.96). In terms of BMI, those who were underweight (reference category) were at higher risk of death. In the multivariable model pancreatic status was not associated with death.

**Figure 2:**
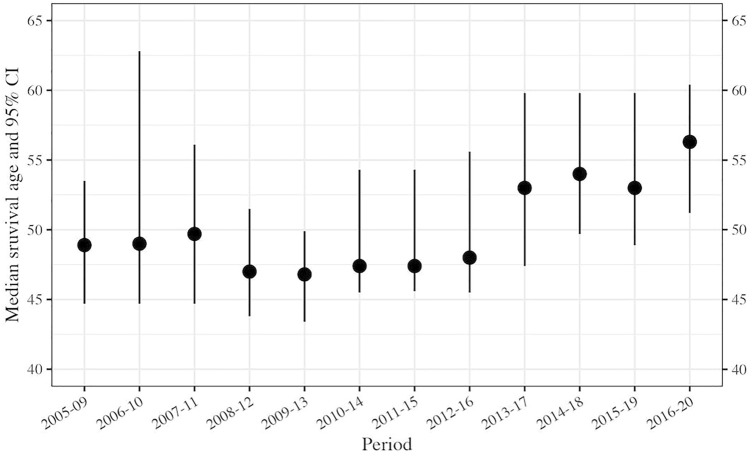
Median survival of people with cystic fibrosis in Australia (5-year cohorts), 2005–2020. Black circles indicate median age data. Error bars represent 95% confidence intervals data. Summary statistics are provided in Table 3.

Table 3 (represented in Fig. 2), shows that the estimated median 5-year survival has increased over a 5-year period from 48.9 (95% CI: 44.7–53.5) years for people born in 2005–2009, to 56.3 (95% CI: 53.0–60.4) years for those born in 2016–2020.

**Table 3:** Median survival of people with CF in Australia (2005–2020).

Period^1^	Year	Median Age, years	95% CI	N at risk	N death
2005–2009	2009	48.9	44.7 – 53.5	3,002	129
2006–2010	2010	49.0	44.7 – 62.8	3,101	128
2007–2011	2011	49.7	44.7 – 56.1	3,196	139
2008–2012	2012	47.0	43.8 – 51.5	3,285	159
2009–2013	2013	46.8	43.2 – 49.9	3,365	180
2010–2014	2014	47.4	45.5 – 54.3	3,402	171
2011–2015	2015	47.4	45.6 – 54.3	3,479	170
2012–2016	2016	48.0	45.5 – 55.6	3,542	176
2013–2017	2017	53.0	47.4 – 59.8	3,568	169
2014–2018	2018	54.0	49.7 – 59.8	3,707	166
2015–2019	2019	53.0	48.9 – 59.8	3,772	171
2016–2020	2020	56.3	53.0 – 60.4	3,802	162

1 Refers to the cohort born in this period.

Figure 2 shows median survival of people with CF in Australia. Each dot and line represent the estimated median survival age and 95% CI, respectively, shown from 2005 to 2020.

## Discussion

This study described estimated survival of people with CF in Australia with survival in those born in 2016–2020 emerging as 56.3 years (95% CI: 51.2–60.4) utilising the data from 2005–2020. Our data demonstrates the increase in median estimated survival among Australian people with CF over the past 15 years, with survival estimates that are comparable with international estimates. According to our study findings, the greatest risk factors for worse survival were low lung function and poorer nutritional status, emphasising the need for ongoing targeted interventions that slow the progression of lung disease and improve nutrition.

National CF registries are valuable tools for performing quality survival analyses and have been instrumental in demonstrating improved survival^6^. Many CF registries show the median age at death, supplemented by a graph representing the distribution of ages at death or the time trends in this median age at death. The Canadian, Irish, UK and US registries determine the estimated median age of survival based on the period approach^2,8,16,18^.

Our results present overall and period survival of people with CF in Australia and the factors associated with survival. In 2012, the median age of survival varied between countries: 47.0 years in Australia (95% CI: 43.8–51.5), 49.7 years in Canada (95% CI: 46.1–52.2)^5^, 43.5 years in the UK (95% CI: 37.6–49.9)^19^ and 41.1 years in the US (95% CI: 37.4–43.1)^20^. In recent years the estimated median age of survival in Australia was not dissimilar to that reported by other registries. For example, in 2019 the median age of survival was estimated to be 54.3 years of age in Canada^21^ and the Cystic Fibrosis Foundation Registry Report from the US calculated the predicted median survival age of a child born that year with CF to be 53 years^22^.

Factors associated with worse survival in the Australian CF population include receiving a lung transplant, lower lung function, and low BMI. In contrast to previous studies, an unexpected association between survival and earlier age of diagnosis was observed in our study. This effect could be explained by a potentially higher proportion of those with severe genotypes identified via NBS or presenting very early, the poorer quality of the diagnosis data captured in earlier years of the data registry, or a chance finding.

Sex and pancreatic status were not found to be independently associated with the survival probability. This finding is different from the previously reported registry survival studies^2^. The study by MacKenzie et al^23^ reported that sex, F508del status and increasing age at diagnosis were independently associated with survival in the US, and female sex has long been associated with worse survival in CF compared to males^2,24^. It is possible that the gender gap in Australia is narrowing through improvements in treatment, diagnosis and lung function trajectory in females^25,26^, although further studies are required to confirm this.

Published data suggest that receiving a lung transplant is associated with earlier death^27^. This finding was also observed in our study. Similarly, poorer lung function and lower BMI were also found to be associated with increased odds of mortality^6–8,27–29^. The results of our study and previous studies^6,27,30^ confirm the impact of lung function and nutritional status on the survival of people with CF, showing that severe and moderate pulmonary impairment and undernutrition have a major impact on survival. These findings justify the emphasis on interventions aimed at optimising pulmonary function and nutritional status in CF guidelines and standards of care^31^.

Improving median survival age in CF can be attributed to many different factors including NBS, nutritional interventions, proactive disease surveillance at both individual and population levels (including via the ACFDR), management of respiratory infections, access to novel therapies and improved standards of care^16,32,33^. The impact of NBS on survival estimates would not be evident for several decades until babies screened at birth reached an age where they would be at risk for death. NBS programmes for CF were first implemented in the early 1970s in the Royal Gwent Hospital in UK^34^. In Australia, NBS started in 1981and was progressively implemented across different states/jurisdictions becoming universal in 2001^35^, potentially explaining increased age of survival in recent years. Those identified by NBS have improved nutritional status and growth compared to people who are diagnosed by symptoms later in life^36^, and some studies indicate improved pulmonary function^37^. People identified at an early age benefit from early interventions to optimize nutrition, prevent and treat lung disease, and early monitoring for liver disease and other complications^38^.

CFTR modulator therapies have the potential to reduce symptoms and increase survival for an increasing number of people with CF^6^. In Australia, the first CFTR modulator (ivacaftor) was approved for use from December 2014, with lumacaftor/ivacaftor (Orkambi^R^) tezacaftor/ivacaftor (Symdeko^R^) and elexacaftor/tezacaftor/ivacaftor (Trikafta^R^) available for patients from October 2018, December 2019 and April 2022 respectively^9^. It is too soon to see the impact of these novel therapies on survival although the estimated median age of survival of people with CF in Australia is expected to continue to improve. The Australian CF registry data is an essential tool to evaluate the impact of CFTR modulator therapies on future clinical outcomes including survival long-term^6^.

### Strengths and limitations

The strengths of our study include the large sample size, the longitudinal data within the ACFDR, the consistency of our results across multiple sub-groups and the unified approach to the analysis. There is a very high participation rate at the centre level as well as in the registry as since mid-2019 participating centres receive payment for data submission which results in a comprehensive national picture of the CF population in Australia^11^. In addition, this study is of the longest running cohort of patients diagnosed via NBS^35^.

As the ACFDR does not capture identifiable data, some death and transplant data could not be verified through national linkages. In addition, data pertaining to CF co-morbidities, such as CF-related diabetes and chronic infections with *P. aeruginosa* were not analysed separately, and could influence survival outcomes. Certain data elements were incompletely recorded for the earlier years for this analysis (i.e. microbiology, CF-related diabetes and socioeconomic status) thus we were unable to account for these factors in our analyses. The differentiation of NBS diagnosis and clinical diagnosis based on meconium ileus /failure to thrive in first few weeks is not absolutely clear and could affect the quality of the diagnosis data. A database redesign conducted in 2018, and a new format of the registry will enable better quality and completeness of the registry records and greater accuracy of future analyses arising from the registry data^9,10^.

## Conclusions

We have demonstrated successive improvements in survival among people with CF in Australia over the last 15 years and identified factors influencing survival. Further research is needed to understand the complex interactions between biological and epidemiological factors not examined in this current study on the severity of disease and survival.

The increase in survival and longevity requires an evolution in models of CF care towards prevention and management of age-related comorbidities such as diabetes, metabolic and cardiovascular disease, malignancies and osteoporosis alongside nutritional and respiratory care^39^. Increasing longevity must be coupled with increasing quality of life and the ability for people with CF to actively participate in a broad range of personal, community and work-based activities. Accordingly, these survival data presented highlight the need for future care models to incorporate a greater focus on the psychological, social, educational and occupational potential of people with CF as they live into old age.

## Data Availability

The datasets generated during and/or analysed during the current study are available from the corresponding author on request and pending approval to the ACFDR Data Access and Research Publishing Committee.
